# Biological effects and mechanisms of fisetin in cancer: a promising anti-cancer agent

**DOI:** 10.1186/s40001-023-01271-8

**Published:** 2023-08-25

**Authors:** Chenhui Zhou, Yi Huang, Sheng Nie, Shengjun Zhou, Xiang Gao, Gao Chen

**Affiliations:** 1 School of Medicine, Zhejiang University, Hangzhou, 310009 China; 2 Department of Neurosurgery, Ningbo First Hospital, Ningbo, 315300 China; 3 Department of Neurosurgery, School of Medicine, Second Affiliated Hospital, Zhejiang University, Hangzhou, China

**Keywords:** Fisetin, Cancer, Anticancer therapy, Biological function, Signal pathways

## Abstract

Fisetin, a natural flavonoid, possesses numerous biological activities that have been extensively studied in various diseases. When it comes to cancer, fisetin exhibits a range of biological effects, such as suppressing cell growth, triggering programmed cell death, reducing the formation of new blood vessels, protecting against oxidative stress, and inhibiting cell migration. Moreover, fisetin has the ability to enhance the effectiveness of chemotherapy. The anticancer properties of fisetin can be attributed to a diverse array of molecules and signaling pathways, including vascular endothelial growth factor (VEGF), mitogen-activated protein kinase (MAPK), nuclear factor-kappa B (NF-κB), PI3K/Akt/mTOR, and Nrf2/HO-1. Consequently, fisetin holds promise as a therapeutic agent for anticancer treatment. In this review, we place emphasis on the biological functions and various molecular targets of fisetin in anticancer therapy.

## Introduction

Cancer poses a significant global health challenge due to its high mortality rates [1, 2]. The development of cancer involves complex processes, including excessive cell proliferation, evasion of apoptosis, sustained angiogenesis, increased migration, and invasion [3, 4]. Unfortunately, current therapies are often expensive, harmful to normal cells, and only provide limited improvements in survival and symptom reduction. Despite advancements in medical technology and surgical techniques, the overall prognosis for many cancer patients remains poor. As a result, there is a pressing need to target anticancer therapy as a crucial area of medical research. Over the past few decades, an increasing number of researchers have focused on unraveling the underlying mechanisms of cancer and exploring new and effective treatment approaches.

Many centuries ago, Hippocrates, often referred to as the father of medicine, famously stated, “Let food be thy medicine and medicine be thy food.” The use of plants for medicinal purposes has been practiced for thousands of years [5]. Flavonoids, which are naturally occurring compounds found in vegetables and fruits, have been extensively studied and shown to have beneficial effects in various diseases, including cancer [6, 7]. Due to their effectiveness, affordability, safety, and the convenience of oral administration, flavonoids have attracted significant research attention to explore their biological activities and underlying mechanisms [8]. Among these flavonoids, fisetin has emerged as a promising candidate. Fisetin, also known as 3,30,40,7-tetrahydroxyflavone, is a natural flavonoid with a well-defined chemical structure. It is widely present in a variety of vegetables and fruits, ranging from 2 to 160 μg/g, including, cucumber, persimmon, strawberry and apple [9]. Fisetin possess diverse biological activities, including inhibiting oxidative stress, anti-inflammation, neuroprotection and anticancer properties [10]. The effects of fisetin have been demonstrated in various types of cancers (Table 1).

**Table 1:** The function and mechanisms of fisetin in cancer

cancer	Function	Signaling pathway
Liver cancer	Resist proliferation, migration and invasion, induce apoptosis, attenuate ROS and inflammation	VEGF, MAPK, caspase 3
Lung cancer	Promote apoptosis and ER stress, induce autophagic cell death, induce mitotic catastrophe, enhance chemotherapeutic effects, resist proliferation, suppress migration and invasion	PI3K/AKT/mTOR, Caspase 3, TRAIL, MAPK
Oral cancer	Induce apoptosis and autophagy, promote ER stress and ROS, suppress proliferation	cytochrome c, AIF and ENDO G, caspase 3, Met/Src
Gastric cancer	Reduce proliferation, induce apoptosis and ROS	CDK, ERK
Ovarian cancer	Induce apoptosis, reduce proliferation	
Prostate cancer	Inhibit the viability and colony formation, promote apoptosis, enhance chemotherapeutic effects, induce autophagic cell death, resist proliferation	β-tubulin, PI3K/Akt, JNK, mTOR, TRAIL, YB-1, MMP-2/9
Breast cancer	Promote apoptosis and invasion and metastasis, enhance chemotherapeutic effects, induce autophagy	PI3K/Akt/mTOR, PARP. HO-1, caspase-3, PTEN/Akt/GSK3β, HER2/neu, PKC/ROS/MAPK, p53, caspase-7, -8 and -9
Colorectal cancer	Enhance radiosensitivity, induce apoptosis, suppress proliferation, resist angiogenesis	AKT, COX, Wnt/EGFR/NF-κB, p53
Osteosarcoma	Inhibit proliferation, enhance chemotherapeutic effects	
Renal cancer	Induce apoptosis, inhibit proliferation and invasion	P53, TET1, 5hmC
Pancreatic cancer	Enhance chemotherapeutic effects, induce autophagy	ERK-MYC, AMPK/mTOR
Cervical cancer	Induce apoptosis, inhibit migration and invasion	Caspase-3/8, ERK1/2
Glioma	Suppress proliferation, inhibit invasion and migration	MAPK
Laryngeal carcinoma	Inhibit proliferation, induce apoptosis and autophagy	ERK1/2, AKT/NF-Κb/mTOR
Bladder cancer	Inhibit proliferation, induce apoptosis	P53, NF-κB
Nasopharyngeal carcinoma	Prevent migration and invasion	LMP1
Leucocythemia	Inhibit proliferation, induce apoptosis and cell cycle arrest, enhance chemotherapeutic effects	Caspase 3, JAK/STAT, MAPK, NO
Skin cancer	Reduce proliferation, inhibit inflammation, induce ROS, have anti-invasive and anti-metastatic effects, enhance chemotherapeutic effects, induce apoptosis and ER stress	P53, p21, PI3K/AKT/mTOR, NF-κB, Nrf2, MAPK, p70S6K,PARP, Wnt/β-catenin

Fisetin exhibits several beneficial effects in cancer cells, including the suppressing proliferation, inducing apoptosis, reducing of angiogenesis, preventing oxidative stress, inhibiting migration, and enhancing chemotherapeutic effects. Moreover, the anticancer properties of fisetin are attributed to the involvement of numerous molecules and signaling pathways, including vascular endothelial growth factor (vegf), mitogen-activated protein kinase (MAPK), nuclear factor-kappa B (NF-κB), PI3K/Akt/mTOR and Nrf2/HO-1. This review summarizes the functions and mechanisms of fisetin as a therapeutic agent in anticancer therapy.

## Biological function of fisetin (Fig. 1)

**Fig. 1:**
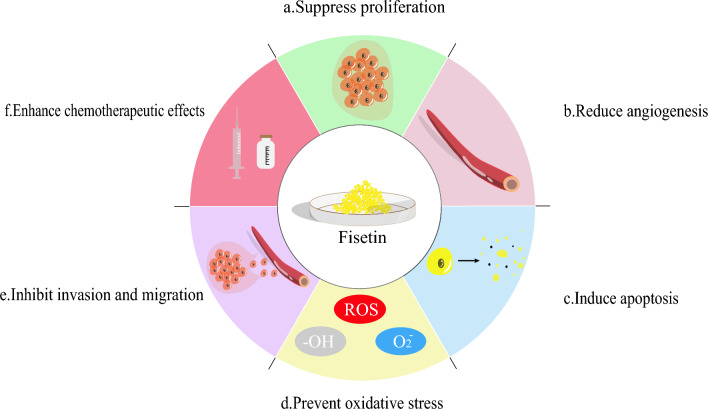
Biological function of fisetin

### Suppress proliferation

Proliferation plays a central role in the progression of cancer and contributes to cancer invasion, metastasis, and relapse [11]. Inhibiting proliferation is a promising strategy for anticancer therapy. The inhibition of proliferation involves various mechanisms, including preventing cells from undergoing mitosis, arresting the cell cycle, and inducing the Warburg effect. Additionally, the regulation of growth factors and activation of cell adhesion molecules are crucial for controlling proliferation. Moreover, multiple signaling pathways and molecules are involved in the process of proliferation in cancer, including MAPK, ERK, VEGF, PI3K/AKT/mTOR and MMPs [12].

The anticancer effects of fisetin on proliferation have been reported in various types of cancers, including astrocytes, oral cancer, gastric cancer, renal carcinoma, and others [13]. Fisetin’s antiproliferative activity is attributed to its ability to suppress DNA synthesis and induce G0/G1 cell cycle arrest in cancer cells [14]. Specifically, fisetin has been shown to inhibit the CDK6/cyclin D complex, which plays a critical role in the G1 phase of the cell cycle. Furthermore, fisetin can induce premature initiation of chromosome segregation and exit from mitosis without normal cytokinesis in cancer cells. Additionally, fisetin interferes with the ligand binding domain of androgen receptor (AR), resulting in a reduction in cancer cell growth. Various signaling pathways are involved in fisetin’s suppression of proliferation in cancer cells. For example, fisetin inhibits the phosphorylation of AKT and MAPK in colon cancer, which are both implicated in cancer cell proliferation.

### Reduce angiogenesis

Angiogenesis is a crucial process involving the formation of new blood vessels from pre-existing vessels within cancerous growth. It represents a significant step in cancer development. Targeting angiogenesis and reducing the formation of new blood vessels is an effective strategy that has shown success in numerous types of cancer cells [15]. In many cancers, the activation of proteases and signaling pathways by specific mediators plays a vital role in driving angiogenesis. These mediators include VEGF, EGF, PDGF, TGF-β, wingless, Wnt signaling, and others [16]. Among these molecules, VEGF is considered the most important factor for angiogenesis in cancer cells [17].

Fisetin is found to have powerful antiangiogenic potential by blocking neovascularization and disrupting the utility of existing blood vessels [18]. Fisetin exhibits its antiangiogenic role by regulating a number of important angiogenesis-related factors in cancer cells, such as VEGF, MMP2/9, eNOS, wingless and Wnt-signaling. VEGF-caused angiogenic response in cancer cells can be strongly inhibited by fisetin [10]. In prostate cancer, fisetin inhibits angiogenesis by reducing NF-κB activity and VEGF expression. Fisetin also inhibits hyaluronan synthesis to upregulate the expression of high-molecular-mass-HA, an antiangiogenic protein [13]. In addition, fisetin can inhibit endothelial cells proliferation and invasion, which are significant in angiogenesis.

### Induce apoptosis

Cell apoptosis, a programmed cell death process, plays a critical role in tumor formation and can be initiated through either the extrinsic pathway or the intrinsic pathway [19]. The extrinsic pathway is triggered by the interaction between death receptors and specific ligands, while the intrinsic pathway involves stimuli that act directly on mitochondria [20]. In both pathways, caspases, which are protease enzymes, play crucial roles and ultimately lead to the cleavage of substrates and fragmentation of DNA. Cancer cells often evade apoptosis by upregulating anti-apoptotic proteins and downregulating pro-apoptotic proteins. Therefore, a therapeutic agent that can sensitize cancer cells to undergo apoptosis represents a promising approach for anticancer therapy [21].

Multiple studies have demonstrated that fisetin has the ability to induce apoptosis in cancer cells, and various mechanisms are involved, including the activation of MAPK, NF-κB, p53, and the generation of reactive oxygen species (ROS) [22]. Fisetin treatment leads to increased expression of pro-apoptotic proteins (Bak, Bax, and Bad), decreased expression of anti-apoptotic proteins (Bcl-xl, Bcl-2, and Mcl-1), and the release of cytochrome c [23, 24]. Fisetin also activates caspase 3/8 and calpain, leading to DNA fragmentation and apoptosis in cancer cells [25]. In bladder cancer, fisetin disrupts mitochondrial integrity and initiates the intrinsic pathway to induce apoptosis. Similarly, fisetin induces mitochondrial-mediated apoptosis in lung cancer by generating ROS, which triggers mitochondrial membrane depolarization, cell apoptosis and DNA fragmentation. The ERK1/2 and NF-κB pathways play pivotal roles in the anti-apoptotic effects of fisetin. In cervical cancer, fisetin could induce apoptosis through the phosphorylation of ERK1/2. In cancer cells, fisetin interferes with NF-κB signaling, resulting in the reduction of survivin, TRAF1, Bcl-xl, Bcl-2, and IAP1/2 levels, ultimately inhibiting apoptosis [8]. In colon cancer, fisetin inhibits COX2 expression, leading to the down-regulation of PGE2 secretion and inactivation of β-catenin, thereby inducing apoptosis. Moreover, fisetin markedly induces apoptosis in renal carcinoma through increased expression of DR5, which is regulated by p53.

### Prevent oxidative stress

Oxidative stress primarily occurs due to the high expression of reactive oxygen species (ROS), including H2O2, hydroxyl radicals, and superoxide anions. ROS are byproducts of cellular metabolism and are normally present at low levels in physiological conditions [26]. However, when ROS levels become elevated due to pathological factors, it triggers oxidative stress, which can damage cellular structures and contribute to the development of cancer and other diseases [27]. The ideal approach for antioxidants is to minimize ROS expression in cancer cells while preserving the physiological effects of ROS [28].

Fisetin demonstrates promising antioxidant properties in cancer [29]. It has the ability to reverse the decreased expression of endogenous antioxidants, thereby maintaining cellular redox homeostasis and neutralizing ROS from various sources [30]. Fisetin reduces the levels of hydroxyl radicals and superoxide anions, with mitochondrial origin being a common source of their generation [31]. Additionally, fisetin treatment can rescue the decrease in cell viability and the increase in ROS production caused by hydrogen peroxide. Furthermore, fisetin possesses antioxidant properties that enable it to counteract the adverse effects of chemotherapy. A previous study indicated that fisetin mitigated cisplatin-induced nephrotoxicity by inhibiting oxidative stress and scavenging free radicals [32].

### Inhibit invasion and migration

Oncogenesis is characterized by the aggressive invasive and migratory abilities of cancer cells [33]. The high invasiveness of cancer cells contributes to the process of cancer metastasis and malignant transformation [34]. Once cancer cells metastasize to other organs in the body, treatment becomes challenging, and it becomes a leading cause of high mortality [35]. Matrix metalloproteinases (MMPs), which are responsible for the degradation of the extracellular matrix, play a critical role in regulating the metastasis of cancer cells. Furthermore, several signaling pathways, including MAPK, NF-κB, PI3K/AKT/mTOR, and EGFR, are known to be involved in the invasion and migration of cancer cells [36, 37].

The inhibitory effects of fisetin on invasion and migration have been demonstrated in various types of cancer cells [38]. For instance, fisetin has been shown to inhibit the metastasis of PC3 prostate cancer cells by reducing the activity of the PI3K/AKT and JNK pathways, resulting in the suppression of MMP-2 and MMP-9 expression [39]. In astrocytoma, fisetin can inhibit cell migration and reduce focal adhesion kinase (FAK) phosphorylation levels, which play a significant role in cell spreading and migration processes. Furthermore, fisetin significantly suppresses the invasion of U-2 cells by decreasing the expression of NF-κB, urokinase-type plasminogen activator (uPA), FAK, and MMP-2/9 [40].

Furthermore, reactivated epithelial–mesenchymal transition (EMT) often facilitates the metastatic spread of cancer cells, which is a process where fully differentiated epithelial cells undergo a transition to poorly differentiated and migratory mesenchymal cells [41, 42]. Fisetin has been shown to have the ability to reverse EMT, thereby inhibiting the invasion and migration of cancer cells [43].

### Enhance chemotherapeutic effects

Chemotherapy is a cornerstone of anticancer treatment [44]. However, it is often associated with unwanted toxic effects and the development of chemoresistance, leading to a poor prognosis [45]. To address these challenges, researchers have investigated the combination of phytochemicals with chemotherapeutic agents, which has shown promising results in enhancing the efficacy of anticancer therapy. By combining phytochemicals with conventional chemotherapy, it is possible to achieve higher treatment efficiency and reduce the required drug doses, thereby minimizing toxic effects. This approach has the potential to prolong survival and improve the quality of life for cancer patients [46, 47].

It has been demonstrated that fisetin has the ability to enhance the effects of chemotherapy [48]. When combined with chemotherapeutic agents, fisetin improves the anticancer effects compared to using either agent alone [49]. For example, Eiman et al. showed combining fisetin with cabazitaxel synergistically inhibited proliferation and metastasis, and promoted apoptosis, thereby enhancing the overall anticancer activities. In lung cancer, the combination of fisetin with carnosic acid demonstrated a stronger effect in regulating apoptosis-related proteins, increasing caspase-3/8/9, Bax, DR, and p53, while decreasing Bcl-2 and Bcl-xl [50]. Fisetin has also been found to enhance the pro-apoptotic effects of cisplatin in teratocarcinoma, as well as sensitize resistant lung cancer and breast cancer cells to cisplatin [49, 51]. Tripathi et al. reported that the combination of fisetin with cisplatin resulted in a fourfold increase in anticancer potential compared to individual treatments [52]. Furthermore, fisetin could arrest cell cycle by increasing cell proportion in G2/S-phase and reducing cell proportion in G2-phase when combined with etoposide [53]. Importantly, numerous studies have shown that fisetin can exert its biological activities without inducing any significant toxic effects [54, 55].

## Signaling pathways of fisetin (Fig. 2)

**Fig. 2:**
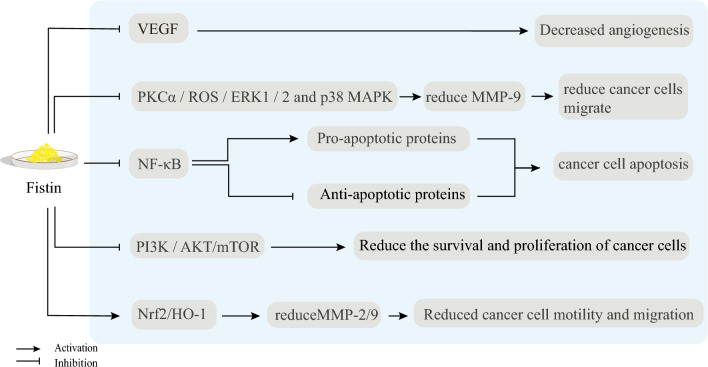
Signaling pathways of fisetin

### VEGF

Endothelial cells play a crucial role in the process of angiogenesis [56]. Vascular endothelial growth factor (VEGF) is a key proangiogenic factor that activates endothelial cells and promotes the formation of new blood vessels in cancer cells [57]. In addition to its role in stimulating angiogenesis, VEGF also regulates cancer cell growth, making it an attractive target for anticancer therapy [58]. Moreover, higher levels of VEGF expression are associated with more advanced stages of cancer and poorer prognosis in cancer patients [59, 60]. Therefore, the development of a non-toxic agent that inhibits VEGF is of great importance in anticancer therapy, as it can help control cancer cell growth and migration.

Fisetin has been shown to effectively inhibit VEGF and reduce its expression, thereby inhibiting angiogenesis and proliferation [61]. In breast cancer and colorectal cancer, fisetin suppresses angiogenesis by decreasing both the expression and release of VEGF from cancer cells [10]. The antiproliferative effect of fisetin, which involves arresting cell cycle in G1 phase and inducing a mild G2/M arrest, is attributed to its inhibitory effect on VEGF expression.

### MAPK

MAPK is a signaling pathway that includes ERK1/2, p38, and JNK1/2, which can be activated through specific phosphorylation cascades [62, 63]. The activation of MAPK is crucial for mediating cancer cell proliferation, apoptosis, and invasion [64]. Phosphorylation of ERK1/2 can result in cell cycle arrest and induction of apoptosis [64, 65]. There is a delicate balance between ERK1/2 and JNK/p38, which plays a critical role in determining whether cancer cells undergo apoptosis or survival [66].

It has been demonstrated that fisetin has the ability to suppress ERK1/2 activation and activate JNK/p38 pathways [67, 68]. The MAPK pathway plays a significant role in the apoptosis process and endoplasmic reticulum (ER) stress induced by fisetin in cancer cells [29]. Fisetin inhibits cell proliferation by targeting the ERK-dependent signaling pathway in astrocytes and gastric cancer [14, 69]. In breast cancer cells, fisetin reduces the expression of MMP-9 by inhibiting PKCα/ROS/ERK1/2 and p38 MAPK activation, thereby leading to a decrease in cell migration [70]. Furthermore, fisetin has been found to regulate MAPK signaling to inhibit cell proliferation and induce apoptosis in acute promyelocytic leukemia cells [71].

### NF-κB

NF-κB is a heterodimeric transcription factor that is involved in various pathological processes in cancer cells [72, 73]. In addition to its role in inflammation, NF-κB regulates important cellular processes such as proliferation, apoptosis, invasion, and angiogenesis [74–77]. NF-κB acts as a positive regulator of growth factors, controlling cell proliferation [78]. Moreover, NF-κB regulates several chemokines that play crucial roles in metastasis and angiogenesis, including IL-8, VEGF, MMPs, and other small molecules [79, 80]. Consequently, NF-κB activity can promote metastasis and angiogenesis. Furthermore, NF-κB has been shown to regulate Bcl-2, an anti-apoptotic protein, thus inhibiting programmed cell death [81]. Given these roles, NF-κB has been identified as a key therapeutic target in cancer, and agents that can inhibit NF-κB activity are considered important anticarcinogens.

The ability of fisetin to suppress NF-κB activity has been demonstrated in various diseases [82, 83], and in the context of cancer, fisetin exerts multiple biological effects by inhibiting NF-κB activity [84]. Fisetin induces apoptosis in cancer cells by inhibiting NF-κB activity, which leads to increased expression of pro-apoptotic proteins and decreased expression of anti-apoptotic proteins [29]. In prostate cancer cells, fisetin enhances the apoptotic effects of tumor necrosis factor-related apoptosis-inducing ligand (TRAIL) by inhibiting NF-κB activity [29]. Fisetin also inhibits the invasion of melanoma and cervical cancer cells by interrupting NF-κB activity [29, 85]. Furthermore, fisetin’s inhibitory effects against bladder cancer are associated with the inhibition of NF-κB, ultimately leading to the suppression of cancer cell proliferation [86].

### PI3K/Akt/mTOR

The PI3K/AKT pathway is closely related to apoptosis in cancer, and inhibiting this pathway can block proliferation and promote programmed cell death (PCD) of cancer cells [87, 88]. Moreover, PI3K/AKT pathway is associated with chemoresistance [89]. As a critical component of the PI3K/AKT/mTOR pathway, mammalian target of rapamycin (mTOR) is also a key factor in anticancer therapies [88]. AKT is phosphorylated upon activation of PI3K, and the activated AKT regulates the phosphorylation of downstream protein mTOR. Consequently, this signaling pathway regulates various cellular processes, including proliferation, apoptosis, differentiation, autophagy, and migration of cancer cells [90–94]. Additionally, the activation of the PI3K/AKT/mTOR pathway is correlated with poor prognosis in several types of cancer. Hence, the three signaling proteins, PI3K, AKT, and mTOR, represent attractive targets for anticancer therapy.

Fisetin has been shown to be effective against PI3K expression, AKT phosphorylation, and mTOR activation in various cancer cells, including prostate cancer, melanoma, breast cancer and colorectal cancer [38]. By suppressing the PI3K/AKT, fisetin reduces cell survival and proliferation in cancer cells [38]. Fisetin also reduces the formation of mTOR, thereby exhibiting its anticancer biological activities [10]. Furthermore, the role of fisetin in decreasing the invasion and migration of lung cancer cells is dependent on the PI3K/Akt/mTOR pathway, and inhibition of these signaling genes abolishes this effect of fisetin [7]. These studies demonstrate that fisetin has the ability to inhibit the PI3K/Akt/mTOR pathway and may serve as a promising therapeutic agent for anticancer therapies.

### Nrf2/HO-1

The activation of the Nrf2/HO-1 pathway plays a significant role in tumor progression and contributes to various aspects of cancer, including cell growth, oxidative stress, angiogenesis, metastasis, chemoresistance, and poor prognosis [95–97]. As a result, agents that can inhibit the Nrf2/HO-1 axis are now considered promising approaches for anticancer therapy [98].

The effects of fisetin on the activation of Nrf2 and upregulation of HO-1 have been demonstrated in various diseases [99, 100]. Notably, Nrf2 is essential for fisetin-mediated upregulation of HO-1 levels, and silencing Nrf2 can block the process of fisetin-induced HO-1 upregulation [101]. In the context of cancer cells, fisetin exhibits anticancer properties by modulating the Nrf2/HO-1 axis. For instance, in breast cancer, fisetin can reduce the expression of MMP-2/9 by upregulating Nrf2 expression and promoting HO-1 transcription, leading to a decrease in cancer cell motility and migration [102]. Fisetin has also been found to induce apoptosis and enhance the effects of chemotherapy through Nrf2/HO-1 pathway [68]. Additionally, the Nrf2/HO-1-mediated oxidative stress response plays a role in the growth inhibitory effects of fisetin [31].

## Absorption and bioavailability of fisetin

It has been demonstrated that fisetin had anticarcinogenic activity by numerous studies [8, 10]. However, the low water solubility of fisetin poses a significant challenge for its administration, which can limit its biological effects [50]. To address this issue and optimize the delivery of fisetin to cancer cells, researchers have explored the use of nanoemulsion formulations. Nanoemulsion is known for its ability to encapsulate hydrophobic active molecules, offering advantages such as small particle sizes, high drug solubility and loading, good stability, sustained drug release, and low toxicity [103]. It serves as an ideal vehicle for improving the efficacy of various anticancer drugs. Fisetin nanoemulsion has shown remarkable improvements in its anticancer potency through extended release, higher drug loading, and enhanced bioavailability. Compared to free fisetin, fisetin nanoemulsion has demonstrated a 3.9-fold increase in the generation of reactive oxygen species (ROS) and induction of apoptosis, highlighting its enhanced efficacy [104].

Another promising approach for improving the bioavailability and therapeutic efficacy of fisetin is liposomal encapsulation. Liposomes are artificial vesicles composed of phospholipids that can encapsulate fisetin molecules. This encapsulation process enhances the stability and solubility of fisetin, allowing for improved delivery and increased bioavailability. Liposomal encapsulation has shown potential in enhancing the anticancer therapeutic effects of fisetin [105].

## Future prospects of fisetin

### Autophagy

Recent research indicates that autophagy reduces the resistance to chemotherapy and radiation [106, 107]. However, there is limited research on the effects of fisetin on autophagy in cancer, and the results are inconsistent. Some studies have shown that fisetin induces autophagic programmed cell death in prostate cancer, while in liver cancer, fisetin has been found to inhibit autophagy [10]. Furthermore, the specific signaling pathways involved in fisetin-mediated autophagy are still not well understood [68]. For example, Suh et al. demonstrated that fisetin-induced autophagy occurs through the AMPK/mTOR pathway, whereas another study found that fisetin-induced autophagy in response to ER stress occurs via an AMPK-independent pathway.

Indeed, it is crucial to clarify the effects and mechanisms of fisetin on cancer cells for the development of effective anticancer therapies. Understanding how fisetin influences key cellular processes such as proliferation, apoptosis, angiogenesis, migration, and autophagy can provide valuable insights into its potential as an anticancer agent. By unraveling the underlying molecular pathways and signaling mechanisms through which fisetin exerts its effects, researchers can identify specific targets for therapeutic intervention and design more targeted treatment strategies. Furthermore, gaining a comprehensive understanding of fisetin’s actions in cancer cells will contribute to optimizing its use in anticancer therapy and maximizing its therapeutic benefits.

## Conclusion

In this review, we have presented compelling evidence for the biological functions of fisetin in anticancer therapy. Additionally, we have discussed various molecular targets of fisetin in cancer. The above findings prompt that fisetin holds promise as a therapeutic agent in anticancer therapy, particularly when combined with other drugs to overcome chemoresistance and enhance efficacy. However, further studies are needed to fully elucidate the complete range of fisetin’s biological functions and underlying mechanisms. Ultimately, fisetin shows great potential for clinical application in anticancer therapy.

## Data Availability

Not applicable.
